# Hibiscus Acid and Chromatographic Fractions from *Hibiscus Sabdariffa* Calyces: Antimicrobial Activity against Multidrug-Resistant Pathogenic Bacteria

**DOI:** 10.3390/antibiotics8040218

**Published:** 2019-11-11

**Authors:** Lizbeth Anahí Portillo-Torres, Aurea Bernardino-Nicanor, Carlos Alberto Gómez-Aldapa, Simplicio González-Montiel, Esmeralda Rangel-Vargas, José Roberto Villagómez-Ibarra, Leopoldo González-Cruz, Humberto Cortés-López, Javier Castro-Rosas

**Affiliations:** 1 Instituto Tecnológico de Celaya, Campus I, Antonio García Cubas Pte. #600 esq. Av. Tecnológico, Celaya C. 3810, Mexico; lizbeth_portillo07@hotmail.com (L.A.P.-T.); aurea.bernardino@itcelaya.edu.mx (A.B.-N.); leopoldo.gonzalez@itcelaya.edu.mx (L.G.-C.); 2 Área Académica de Química, Instituto de Ciencias Básicas e Ingeniería, Ciudad del Conocimiento, Universidad Autónoma del Estado de Hidalgo, Carretera Pachuca-Tulancingo Km. 4.5, Mineral de la Reforma C.P 42183, Mexico; cgomeza@uaeh.edu.mx (C.A.G.-A.); gmontiel@uaeh.edu.mx (S.G.-M.); jrvi@uaeh.edu.mx (J.R.V.-I.); b_et_ocl@hotmail.com (H.C.-L.)

**Keywords:** anti-microbial, Hibiscus acid, *Hibiscus sabdariffa*, multidrug-resistant, antibiotic

## Abstract

The anti-microbial properties of acetone extracts from *Hibiscus sabdariffa* calyces, fractions isolated by silica gel chromatography and hibiscus acid purified from some of these fractions and additionally identified by nuclear magnetic resonance spectroscopy, mid-infrared spectroscopy and X-ray diffraction, were studied against both multidrug-resistant *Salmonella* strains and pathogenic *Escherichia coli* bacteria. Gel diffusion was used to determine the anti-microbial effects. The mode of action of hibiscus acid was determined by crystal violet assay. Hibiscus acid and 17 of the 25 chromatographic fractions obtained, displayed an anti-microbial effect against all bacterial strains tested. Hibiscus acid showed a greater anti-microbial effect than the acetone extract against most of the bacteria strains, while chromatographic fractions IX–XIV exerted the greatest anti-microbial effect against all bacteria. The minimum inhibitory concentration of the acetone extract was 7 mg/mL, and the minimum bactericidal concentration was 10 mg/mL, while the corresponding values for hibiscus acid were 4–7 and 7 mg/mL, respectively. The results of the crystal violet assay indicate that hibiscus acid alters membrane permeability. Hibiscus acid is a potential alternative to control multidrug-resistant bacteria. Due to its ready availability and easy extraction from *H. sabdariffa*, hibiscus acid is potentially useful in the food industries.

## 1. Introduction

*Salmonella* is one of the leading causes of foodborne diseases, and its infection (salmonellosis) is spread worldwide. Due to its prevalence, salmonellosis has become a public health burden, representing significant costs in many countries. A range of fresh fruit and vegetables, especially those eaten raw (lettuce, sprouts, melon and tomatoes), are implicated in *Salmonella* infection [1]. *S. enterica* subspecies *enterica* is composed of more than 1500 serotypes with some of great importance, such as S. Typhimurium and *S*. Enteritidis. *S*. *enterica* subsp. *enterica* is responsible for more than 99% of human salmonellosis and therefore it is widely studied [2].

Another relevant group of foodborne bacteria is the diarrheagenic *Escherichia coli* pathotypes, including enterotoxigenic, enteroinvasive, enteroaggregative, diffuse adherent and Shiga toxin-producing strains [3]. Some studies documented the importance of diarrheagenic *Escherichia coli* pathotypes as agents associated with acute and persistent diarrhoea in Mexican children [4,5,6,7]. These *E. coli* strains circulating in Mexico were identified in various food and beverages and diarrheic faecal stool samples [5,8,9,10,11].

The emergence of multidrug-resistant *Salmonella* strains and *E. coli* pathotypes are related to the use of antibiotics in animals. Resistant bacteria can be transmitted to humans through foods, especially those consumed raw or of animal origin [12]. The presence of multidrug-resistant pathogenic bacteria in food is an important public health issue [13].

The increased resistance of pathogenic bacteria to antibiotics has intensified the demand for safe and natural alternative anti-microbial agents in food products. Plant species used for medicinal purpose and human consumption are currently being studied since they may constitute a source of anti-bacterial compounds. It was reported that extracts obtained from calyces of roselle (*Hibiscus sabdariffa*) have an anti-microbial effect on antibiotic-resistant and non-resistant pathogenic microorganisms [14,15,16,17,18,19,20,21,22,23,24]. The extracts from *H. sabdariffa* calyces are a possible alternative to control antibiotic-resistant pathogenic bacteria.

Calyces of *H. sabdariffa* are known to contain chemical compounds, such as organic acids, phytosterols, polyphenols and anthocyanins [25]. It was suggested that different compounds such as anthocyanins, polyphenol or protocatechuic acid are responsible for the anti-microbial activity of *H. sabdariffa* [21,25,26,27]. However, no prior studies fully demonstrated the anti-microbial effect of the specific chemical compounds in *H. sabdariffa* or reported the isolation of specific anti-microbial constituents from its calyces, which are used in many regions of the world in hot and cold beverages. It is possible that in the *H. sabdariffa* calyces there are other compounds other than those suggested and that they are primarily responsible for the anti-microbial activity.

Hydroxycitric acid, hibiscus acid and it derivatives as the major organic acids in the leaves and calyces extracts of *H. sabdariffa* [28].

Hibiscus acid was demonstrated to have an inhibitory effect on pancreatic α-amylase and intestinal *α*-glucosidase, resulting in reduction of carbohydrate metabolism and blood insulin levels [29]. Furthermore, hibiscus acid was demonstrated to have a vasorelaxant effect on the rat’s aorta [30].

Hibiscus acid is not commercially available; however, it is a chiral compound and its diastereomer, garcinia acid from (*Garcinia cambogia*) is commercially available.

The present study sought to isolate and identify at least one chemical compound with anti-microbial activity from *H. sabdariffa* calyces and evaluate its anti-microbial activity against multidrug-resistant foodborne bacteria.

## 2. Materials and Methods

### 2.1. Preparation of Hibiscus Sabdariffa Extract

Ten kilograms of dehydrated calyces of *H. sabdariffa* (“Criolla de Oaxaca” variety) grown in Oaxaca, Mexico were used in the study. The calyces were stored in a closed polyethylene container at room temperature until use. The acetonic extract from calyces of *H. sabdariffa* was obtained exactly as we previously described. Briefly, samples (100 g) of dehydrated calyces were placed in glass flasks and 900 mL of acetone were added (Sigma-Aldrich, Toluca, Mexico). The flasks were hermetically sealed and stored at room temperature for 7 days with manual shaking for 1 min once a day. After, the liquid phase was filtered through filter paper (Whatman Grade 4). The filtered extracts were concentrated in a rotary evaporator (V-800, Vacuum Controller, BÜCHI, Switzerland). The acetone was completely removed from the rotaevaporated concentrate by placing it in an air recirculation oven (Ambi-Hi-Low Chamber, Lab-Line, Jefferson, MO, USA) at 45 °C for 24 h [22,31].

### 2.2. Chromatographic Fractionation of Acetone Extract

Two hundred and thirty grams of dry acetone extract of *H. sabdariffa* calyces was separated by column chromatography. The dried extract was mixed with silica gel (Sigma-Aldrich, Toluca, México), previously activated at 120 °C for 1 h in a drying recirculation oven (Ambi-Hi-Low Chamber, Lab-Line, Jefferson, USA), at a ratio of 1:2. A glass chromatography column was filled with the silica gel–acetonic extract mixture. Different solvents (hexane, hexane–ethyl acetate, ethyl acetate, ethyl acetate–methanol and methanol) were used as the mobile phase to recover consecutive 100 mL fractions from the packed column. The fractions were concentrated on a rotary evaporator, placed in glass vials and analysed by thin-layer chromatography. The fractions whose components showed the same level of displacement in the plate were pooled and placed in an air recirculation oven at 40 °C to evaporate solvent residues. The anti-microbial activity of the chromatographic collections against multidrug-resistant pathogenic bacteria was determined.

### 2.3. Extraction of Hibiscus Acid

Two hundred and thirty grams of dry acetone extract of *H. sabdariffa* calyces was packed with silica gel in a chromatographic column, as described in Section 2.2. Hexane was used as the mobile phase to separate the oils in the extract, and 600 mL fractions were recovered in glass flasks. All the chromatographic fractions obtained were rotary-evaporated to remove the solvents and concentrate the separated compounds. After discarding most of the oils from the extract, the solvent mixture hexane–ethyl acetate (9:1 *v/v*) was used as the mobile phase to remove all residual oils. The mobile phase (8:2 *v/v*) passed through the packed column until some small crystals were observed in the rotary-evaporated fractions and it was then used at a ratio of 7:3 (*v/v*) to obtain well-defined crystals in the rotary-evaporated fractions. The crystals were analysed by thin-layer chromatography to determine their purity, re-crystallised using 7:3 (*v/v*) acetone–ethyl acetate in a separatory funnel and then stored for 24 h. Once the formation of crystals on the wall of the separation funnel was observed, the liquid was decanted, and the crystals were recovered. Finally, the residual acetone was removed in an air recirculation oven at 45 °C for 2 h.

### 2.4. Structural Identification of Hibiscus Acid

#### 2.4.1. Nuclear Magnetic Resonance Spectroscopy

The crystals (Section 2.3) were examined by proton nuclear magnetic resonance (^1^H NMR) spectroscopy, using deuterated acetone (acetone-*d*_6_; Sigma-Aldrich, Toluca, Mexico) to solubilize the crystals, in a 400 MHz NMR spectrometer (Jeol, Tokyo, Japan). The acquired spectra were analysed using MestReNova 2009 software (version 6.0.2-5475; Mestrelab Research S.L., Santiago de Compostela, Spain).

#### 2.4.2. Infrared Spectroscopy with Attenuated Total Reflection

The crystals (Section 2.3) were ground in a mortar to reduce the particle size and analysed using a diamond-accessorised attenuated total reflection infrared spectrometer (Frontier, Perkin Elmer, Norwalk, CT, USA) at 25 ± 2 °C. Infrared spectra were recorded between 4000 and 400 cm^−1^ at a resolution of 4 cm^−1^, and 64 spectra per sample were co-added to improve the sample-to-noise ratio.

#### 2.4.3. X-ray Crystallography

The crystals were grown in aqueous acetone by slow evaporation. Diffraction data were measured on a Gemini CCD diffractometer (Oxford Diffraction Ltd., Abingdon, Oxfordshire, England) at room temperature using graphite-monochromated CuK*α* radiation (*λ* = 1.54184 Å) and processed using the CrysAlis program (version 1.171.33.31, 2009; Oxford Diffraction Ltd., Abingdon, UK). The structure was solved using Olex2 [32]) and SHELXT [33] structure solution program using intrinsic phasing or direct methods and refined with the crystal structure refinement program SHELXL [34] using least-squares minimization.

#### 2.4.4. Differential Scanning Calorimetry

The melting point of hibiscus acid was measured using a differential scanning calorimeter (Q2000, TA Instruments, New Castle, NJ, USA), previously calibrated with indium (onset temperature *T*_o_ = 156.6 °C, transition enthalpy Δ*H* = 28.4 J/g, respectively. Five milligrams of hibiscus acid crystals were placed in an aluminum crucible, which was then sealed and heated from 25 to 250 °C at a rate of 5 °C/min. The transition temperatures and Δ*H* values were obtained directly using Universal Analysis software version 4.4A (TA Instruments).

### 2.5. Determination of the Anti-microbial Effect of Acetone Extract, Chromatographic Collections and Hibiscus Acid

#### 2.5.1. Preparation of Test Solutions

Solutions of acetone extract, chromatographic collections and hibiscus acid were prepared at final concentrations of 100 mg/mL. Only distilled water was used to prepare the solutions of acetone extract and hibiscus acid. To obtain the solutions from the fraction collections, a mixture of distilled water and 20% Tween 80 (Sigma-Aldrich, Toluca, Mexico) was used.

#### 2.5.2. Bacterial Strains

Eight multidrug-resistant bacteria strains were isolated from food as follows: *Salmonella* Montevideo C1 and *S*. Typhimurium C65 from cilantro [24], *S*. Typhimurium C63 from carrots [18], enteroinvasive *E*. *coli* MAC B from nopalitos [16], enteropathogenic *E*. *coli* MAC A from coriander [35], and enterohemorrhagic *E. coli* EHEC A and two strains of Shiga toxin-producing *E*. *coli* C558 and C636 from raw beef, in our laboratory. All bacteria were resistant to the same 10 antibiotics (kanamycin, neomycin, streptomycin, amikacin, tetracycline, erythromycin, chloramphenicol, ceftriaxone, nalidixic acid and trimethoprim/sulphamethoxazole) according to the Clinical and Laboratory Standards Institute (CLSI) criterion [36].

#### 2.5.3. Preparation of Bacterial Strains

The eight antibiotic-resistant strains were inoculated in 3 mL of tryptic soy broth (TSB; Bioxon, Becton Dickinson, Ciudad de México, Mexico) and incubated at 35 ± 2 °C for 18 h. The cultures were washed twice in sterile isotonic saline (0.85% NaCl; ISS) by centrifugation at 3500 rpm for 20 min, and the pellet was resuspended in ISS at approximately 10^9^ colony forming units/mL (CFU/mL). Finally, a decimal dilution of these washed cultures was done with ISS to produce a final approximate concentration of 8 log CFU/mL [22,31].

#### 2.5.4. Anti-microbial Activity of Acetone Extract, Chromatographic Collections and Hibiscus Acid

The gel diffusion technique with paper discs was used as follows: 100 μL washed bacterial cultures, from a concentration of 1 × 10^8^ CFU/mL, were inoculated onto trypticase soy agar plates (Bioxon, Becton Dickinson) and distributed over the agar by the streak plate method. Sterilized paper discs (Whatman Grade 5, 6-mm diameter) were placed on the surface of the inoculated agar. Then, 20 µL aliquots containing acetonic extract, chromatographic collections and hibiscus acid, respectively, were placed on the paper disks (final dose per disk: 2 mg extract, chromatographic collection or hibiscus acid). ISS was used as a negative control. Treatments were performed in triplicate. The plates were incubated at 35 ± 2 °C for 24 h. For each treatment, the diameters (mm) of the resulting inhibition zones were measured and expressed as the average [31].

### 2.6. Minimum Inhibitory Concentration and Minimum Bactericidal Concentration

The broth macrodilution method [37] was used to obtain the minimum inhibitory concentration (MIC) and minimum bactericidal concentration (MBC). Tubes were prepared with TSB containing acetonic extract or hibiscus acid at concentrations of 1–100 mg/mL. The tubes were inoculated with a final suspension of microorganisms at 1 × 10^5^ CFU/mL (from the culture washed in ISS at a concentration of 1 × 10^9^ CFU/mL, 2 decimal dilutions were made in TSB, and from the last dilution 10 µL were taken and inoculated in a tube containing 990 µL to have a final concentration of 1 × 10^5^ CFU/mL) and incubated at 37 °C for 24 h. The MIC was the lowest concentration of acetone extract or hibiscus acid to inhibit bacterial growth without turbidity in the tubes. To assess the MBC, TSB tubes containing the lowest concentrate ions of extract or hibiscus acid and no turbidity were inoculated into trypticase soy agar using the pour plate technique and incubated at 35 °C for 24–48 h. The MBC was defined as the lowest concentration of acetone extract or hibiscus acid that showed no colony growth in TSB.

### 2.7. Measurement of Permeability with Crystal Violet

Alteration of membrane permeability was detected by crystal violet assay exactly as described by Devil et al. 2010 [38]. Briefly, one hundred μL of *S*. Typhimurium C65 and enterohemorrhagic *E. coli* EHEC A were inoculated in TSB and incubated at 37 °C for 6 h. The bacterial suspensions were centrifuged at 10,000 rpm for 20 min. The supernatant was discarded and the cell pellets were washed twice with 0.5 mM potassium phosphate buffer solution (PBS). The bacterial cell suspension was prepared by re-suspending the cell pellet in PBS. The washed bacterial cell suspensions were incubated with different concentration of hibiscus acid at minimum sub-inhibitory concentration (MSIC; 1.25 mg/mL and 1.75 mg/mL for enterohemorrhagic *E. coli* EHEC A and *S*. Typhimurium C65, respectively), MIC (5 mg/mL and 7 mg/mL for enterohemorrhagic *E. coli* EHEC A and *S*. Typhimurium C65, respectively), 10× MIC (50 mg/mL and 70 mg/mL, for enterohemorrhagic *E. coli* EHEC A and *S*. Typhimurium C65, respectively), MBC (7 mg/mL) and ethylenediaminetetraacetic acid (EDTA, positive control, 0.25 M) at 37 °C for 60 min. Control samples were prepared similarly without treatment and EDTA (0.25 M) was used as a positive control. The cells were harvested (10,000 rpm for 5 min) and suspended in PBS containing crystal violet (10 µg/mL). The cell suspension was then incubated (10 min at 37 °C) and centrifuged (10,000 rpm for 5 min). The optical density (OD) 590 of the supernatant was measured using a UV-VIS spectrophotometer (Thermo Scientific, Nanodrop, Verona, Wisconsin, USA). The OD value of crystal violet solution was considered to be 100% excluded. The OD of the supernatant of the normal untreated cell was used as blank. The percentage of crystal violet uptake for all samples was calculated using the following formula:

% uptake of crystal violet = (OD Value of sample)/(OD Value of CV solution) × 100(1)

### 2.8. Statistical Analysis

Significant differences (*p* < 0.05) between treatments were calculated by analysis of variance and Tukey’s test using SPSS Statistics 20 (IBM Corp., Armonk, NY, USA).

## 3. Results and Discussion

### 3.1. Anti-microbial Activity of Acetonic Extract of Hibiscus Sabdariffa

A total of 4.6 g of dry acetonic extract was obtained per 100 g of dehydrated *H*. *sabdariffa* calyces. The dry extract had anti-microbial activity against the eight multidrug-resistant *Salmonella* and pathogenic *E. coli* strains, while the radial inhibition zone on the culture medium varied from 9.8 to 12.6 mm. These results agree with those previously reported on the anti-microbial effect of extracts obtained from *H. sabdariffa* calyces [18].

### 3.2. Anti-microbial Activity of Chromatographic Collections against Pathogenic Bacteria

The acetone extract was separated by column chromatography into 903 fractions using different solvent mixtures (Table 1). Fractions displaying the same or similar thin-layer chromatogram were pooled together. Among these 25 collections (I–XXV; Table 2), 14 were anti-microbial against all multidrug-resistant *Salmonella* and pathogenic *E. coli* strains tested (Table 3), while three showed an effect against some of the *Salmonella* and pathogenic *E. coli* strains (Table 3). Collection VI was only effective against *Salmonella* C1 and C65, and collection VII, only against *Salmonella* C65, respectively. In contrast, collection XXII was not anti-microbial against *E. coli* C558, *E. coli* C636 and enteroinvasive *E. coli* MAC B (Table 3). Finally, collections I, II, III, IV, V, VIII, XXIV and XXV had no anti-microbial effect against any pathogenic bacteria. In general, statistically significant differences were observed between the effects produced by some collections and within collections against different pathogenic strains (Table 3).

In a study on anti-microbial chromatographic collections from plants with solvents of different polarities, Avila-Sosa et al. [39] obtained and fractionated the chloroform extract from Mexican oregano (*Lippia berlandieri* Schauer) using chloroform and mixtures of chloroform–acetone (70:30, *v/v*), chloroform–acetone (30:70, *v/v*), acetone–methanol (70:30, *v/v*) and acetone–methanol (30:70, *v/v*) as mobile phases. Afterwards, they evaluated the anti-microbial activity of the chromatographic fraction collections obtained against *E. coli*. While most of the chromatographic collections showed anti-microbial activity against *E. coli*, the collections higher in polarity were less potent. Consistent with that observation, the low and intermediate polarity collections displayed the greatest anti-microbial effect in the current work (Table 1, Table 2 and Table 3). Furthermore, Kuete et al. [40] determined the methanolic extract of *Ficus polita* (FP) was anti-microbial against *S*. Typhi (ATCC 6539) and two strains of *E. coli* (ATCC 8739 and AG100). In addition, the researchers tested five chromatographic fractions from FP (FPR1–FPR5), obtained using different mobile phases: hexane (FPR1); 75:25 (*v/v*) hexane–ethyl acetate (FPR2); 50:50 (*v/v*) hexane–ethyl acetate (FPR3); ethyl acetate (FPR4); methanol (FPR5). Of the five fractions, only those of low polarity (FPR1 and FPR2) exhibited anti-microbial activity against the studied strains.

Do et al. [41] also investigated the anti-microbial effect of five chromatographic fraction collections obtained from the methanolic extract of *H. sabdariffa*, using different solvent mixtures as mobile phases: 50% hexane–50% ethyl acetate (CF1); 30% hexane–70% ethyl acetate (CF2); 90% ethyl acetate–10% methanol (CF3); 60% ethyl acetate–40% methanol (CF4); 70% ethyl acetate–30% methanol (CF5). Among them, only CF3, which was active against *E*. *coli*, *Staphylococcus aureus*., *Bacillus cereus* and *B*. *subtilis*, and CF4 and CF5, which were active against *S*. *aureus* and *B*. *subtilis*, possessed anti-microbial properties. Moreover, only CF3, which showed the greatest anti-microbial action, contained the flavonoid quercetin, among other unidentified compounds. However, further studies would be required to conclusively identify the anti-microbial molecules in CF3–CF5 and the methanolic extract, and any interactions responsible for the activity.

In the current study, collections IX, X, XI, XIII and XIV showed greater anti-microbial activity than the rest. These collections were obtained with the polarities of the following mixtures: 70% hexane–30% ethyl acetate, 60% hexane–40% ethyl acetate and 50% hexane–50% ethyl acetate (Table 1 and Table 2). It is important to note that defined crystals were formed in the pooled fractions IX, X and XI, which were collected using 70% hexane–30% ethyl acetate as the mobile phase (Table 1 and Table 2).

Since the preliminary NMR analysis of the crystals suggested the presence of hibiscus acid and other compounds, a second chromatographic separation was completed using another sample of dry acetonic extract (230 g) from *H. sabdariffa* to obtain a higher concentration of crystals for purification. The aim was to confirm the presence and anti-microbial activity of hibiscus acid by different structural analysis techniques. The second column chromatography separating the acetonic extract yielded presumptive crystals of hibiscus acid.

### 3.3. Obtaining Presumptive Crystals of Hibiscus Acid from the Acetonic Extract

Following the procedure described in Section 2.3, a total of 413 fractions were grouped into four collections, according to the mobile phase used: hexane (17 fractions), 90% hexane–10% ethyl acetate (52 fractions), 80% hexane–20% ethyl acetate (61 fractions) and 70% hexane–30% ethyl acetate (283 fractions). After purification, 65 g presumptive crystals of hibiscus acid (collection IV, fractions 113–413) were obtained from 230 g acetonic extract of *H. sabdariffa* (1.3% crystals from 5 kg dehydrated calyces) and, additionally, characterized by NMR, infrared spectroscopy and X-ray crystallography, as described in Section 3.4.1, Section 3.4.2 and Section 3.4.3, respectively, to identify the structure of hibiscus acid.

### 3.4. Structural Identification of Hibiscus Acid

#### 3.4.1. ^1^H NMR Spectrum

**Figure 1:**
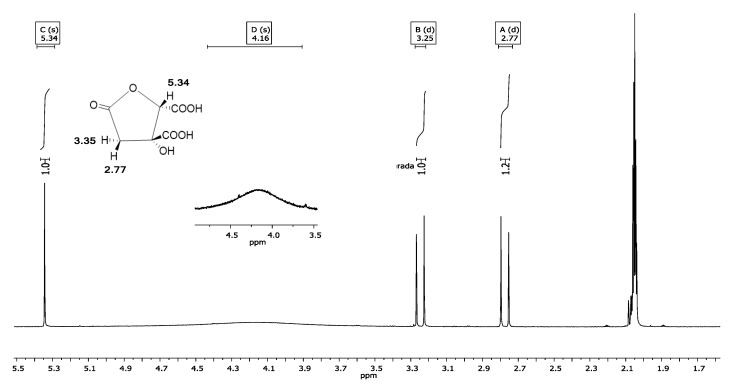
^1^H NMR spectrum at 400 MHz in acetone-*d*_6_ of purified crystals obtained from *Hibiscus sabdariffa* calyces acetonic extract.

The ^1^H NMR spectrum of presumptive crystals of hibiscus acid corresponded to that of the molecular structure of hibiscus acid (Figure 1). Most of the proton signals (^1^H) appeared between *δ*_H_ 0 and 12. The signal observed at *δ*_H_ 2.05 corresponds to the acetone-*d*_6_ used to dissolve the presumptive crystals of hibiscus acid. The other signals were *δ*_H_: 5.34 (^1^H, singlet [s], CH-COOH), 4.16 (^1^H, s, COH-COOH), 3.25 (^1^H, doublet [d], *J* = 17.2 Hz, H*_a_*H_b_C-C=O), 2.77 (^1^H, d, *J* = 17.2 Hz, H_a_H*_b_*C-C=O), where *J* is the coupling constant. Accordingly, the signals of the spectrogram shown in Figure 1 correspond to the molecular structure of hibiscus acid and the deuterated solvent used as the vehicle.

In an NMR (400 MHz) analysis of the structure of hibiscus acid determined using acetone-*d*_6_ to dissolve the crystals, Ibnusaud et al. [42] detected signals at *δ*_H_ 5.38 (^1^H, s, CH-COOH), 3.30 (^1^H, d, *J* = 17.1 Hz, H*_a_*H_b_C-C=O) and 2.80 (^1^H, d, *J* = 17.1 Hz, H_a_H*_b_*C-C=O). In previous ^1^H NMR analysis of hibiscus acid prepared from *H. sabdariffa* calyces extracts, the crystals were dissolved in deuterated water [43], deuterated dimethylsulphoxide [39] and deuterated methanol [40]. The resulting spectrograms showed two doublets at *δ*_H_ 2.88 and 3.41, respectively (*J* = 18.4 Hz) [38] signals at *δ*_H_ 5.31 (^1^H, s), 3.23 (^1^H, d, *J* = 17.19 Hz) and 2.77 (^1^H, d, *J* = 17.18 Hz) [43]; and signals at *δ*_H_ 5.25 (^1^H, s), 3.20 (^1^H, d, *J* = 17.3 Hz) and 2.69 (^1^H, d, *J* = 17.3 Hz) [44]. In this context, the parameters published by Ibnusaud et al. [42] and Rasheed et al. [45] are most similar to those obtained in the present work (Figure 1).

#### 3.4.2. Infrared Spectroscopy

**Figure 2:**
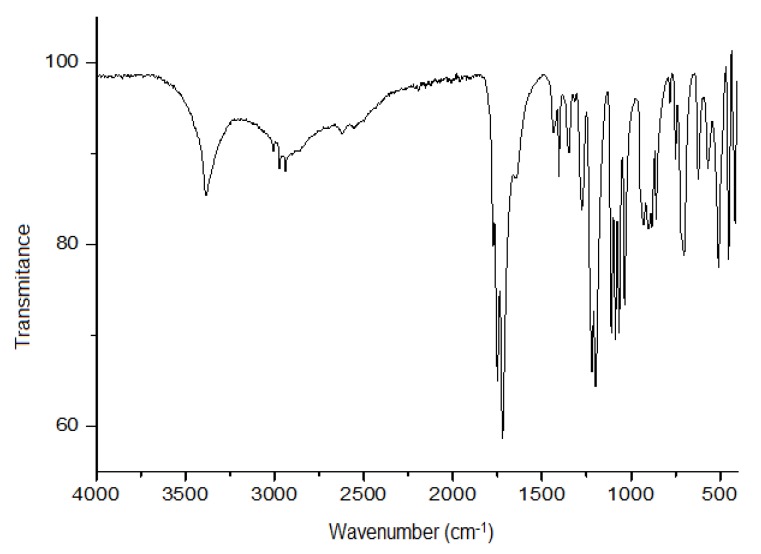
Infrared spectrum of purified crystals obtained from *Hibiscus sabdariffa* calyces acetonic extract.

The infrared spectrum of the crystals prepared from the *H. sabdariffa* acetone extract contained signals at 3410 cm^−1^ (OH groups), 1797 cm^−1^ (ester groups) and 1742 cm^−1^ (C=O stretching) (Figure 2). These values corresponded strongly to those reported by Ibnusaud et al. [42], which were 3400, 1790 and 1735 cm^−1^.

#### 3.4.3. X-ray Crystallography

**Figure 3:**
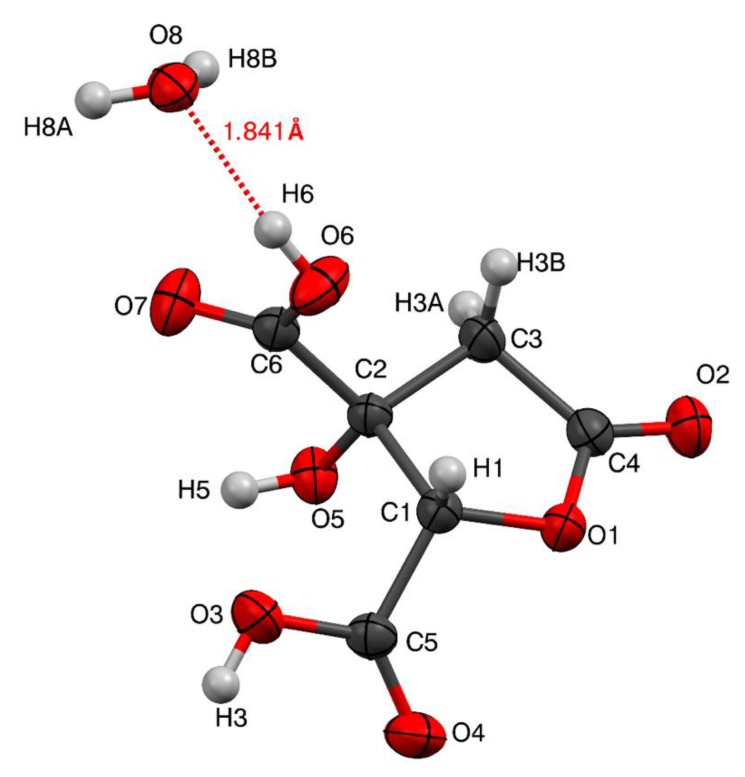
Molecular structure of hibiscus acid isolated from *Hibiscus sabdariffa* calyces acetonic extract determined by X-ray diffraction. Ellipsoids shown at 50% of probability.

The molecular structure of hibiscus acid (Figure 3), was confirmed by X-ray diffraction which was solvated with a water molecule through a hydrogen bond (O6-H6•••O8, distance = 1.841 Å, ∡ O6-H6•••O8 = 162.62°) (Figure 3). Hibiscus acid is a five-membered lactone ring, with four carbon atoms and one oxygen atom. C3 (sp2) has a double-bonded oxygen atom, C1 an OH group and a COOH group, and C2 a COOH group, respectively.

**Figure 4:**
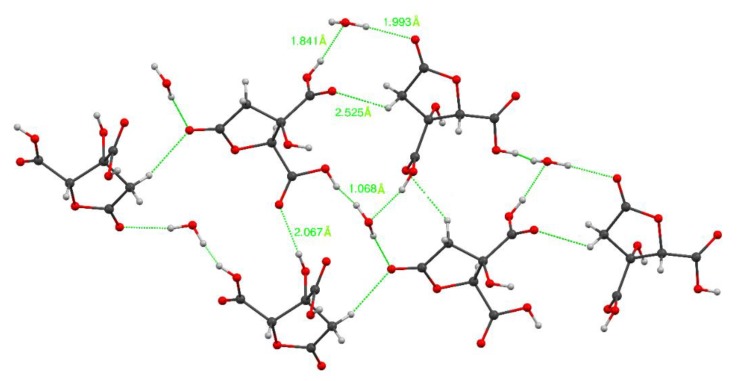
Hydrogen bond contacts of hibiscus acid isolated from *Hibiscus sabdariffa*.

The crystallographic details and refined structure of hibiscus acid are provided in Table 4 and Tables S1–S5. Analogous X-ray crystallographic data of hibiscus acid, albeit attached to a dimethylsulphoxide molecule, were presented by Zheoat et al. [44]. Figure 4 shows the possible hydrogen bond donors of the hibiscus acid molecule, in which the interactions that can be established with other molecules of hibiscus acid and water molecules are observed.

#### 3.4.4. Hibiscus Acid Melting Point by Differential Scanning Calorimetry

The differential scanning calorimetry of the crystals of hibiscus acid showed a *T*_o_ of 186.87 °C, peak temperature of 190.61 °C and a final temperature of 194.15 °C, while the fusion enthalpy (Δ*H*) was 146.7 J/g. Contrastingly, Ibnusaud et al. [37] reported a melting point of 182 ºC for hibiscus acid, which differs from the current results, possibly because of either the equipment or the technique used by the authors.

### 3.5. Anti-microbial Effect of Hibiscus Acid

Hibiscus acid demonstrated an anti-microbial effect against all multidrug-resistant *Salmonella* and pathogenic *E. coli* strains (Table 5). In general, the anti-microbial effect of hibiscus acid was higher (*p* < 0.05) than that of the acetonic extract (Table 5). Previous reports attributed the anti-microbial activity of *H. sabdariffa* calyces to compounds, such as protocatechuic acid and anthocyanins, in the plant [14,21,23,46]. However, no information regarding the isolation and identification of anti-microbial compounds obtained directly from *H*. *sabdariffa* calyces has been published until now.

It should be noted that while several publications described the anti-microbial effect of *H. sabdariffa* calyx extracts obtained with solvents of varying polarities (including acetonic extract) when tested against different pathogenic bacteria, no published article describes or suggests that hibiscus acid or its derivatives are anti-microbial. Furthermore, there is no proof of their efficacy in the control and elimination of multidrug-resistant pathogenic bacteria. In other words, this document constitutes the first report on the anti-microbial activity of hibiscus acid, even against multidrug-resistant pathogenic bacteria. The MIC and the MBC of hibiscus acid were determined to assess its potential use as an anti-microbial agent in the industry.

### 3.6. Determination of the MIC and MBC of the Acetone Extract and Hibiscus Acid

The MICs and MBCs of the acetonic extract and hibiscus acid obtained from *H. sabdariffa* calyces were determined using the eight multidrug-resistant pathogenic strains. For hibiscus acid, the MIC values were 4–7 mg/mL, while the MBC range was 5–7 mg/mL (Table 6). The MIC of the acetonic extract was 7 mg/mL for all the pathogenic strains, while the MBC was 10 mg/mL for most of the pathogenic bacteria (Table 6). Abdallah [42] evaluated the MIC and MBC of the *H. sabdariffa* calyces methanolic extract by broth dilution using five multidrug-resistant *Acinetobacter baumannii* strains and obtained MIC and MBC values of 25–50 and 50–100 mg/mL, respectively. These MIC and MBC values are higher than those obtained with the acetonic extract of *H. sabdariffa* (Table 6).

Yin and Chao [26] tested aqueous and ethanolic extracts of *H. sabdariffa* calyces, obtaining MIC values of 0.112–0.144 and 0.072–0.096 mg/mL, respectively, for *S*. Typhimurium, *E*. *coli*, *Listeria monocytogenes*, *S*. *aureus* and *B*. *cereus*. The MIC of the aqueous extract against *Campylobacter* strains susceptible to antibiotics varied between 0.096 and 0.152 mg/mL [27].

As mentioned above, protocatechuic acid is a compound that was reported to be present in the calyces of *H. sabdariffa* and is likely to be anti-microbial [21,26,27]. However, there is no information available in the literature regarding the isolation, characterization or concentration of the protocatechuic acid in *H. sabdariffa* calyces. Protocatechuic acid is widely distributed in a variety of plants [46]. Commercial protocatechuic acid showed anti-microbial activity against *S. aureus*, *Klebsiella pneumoniae*, *Pseudomonas aeruginosa* and *A*. *baumannii,* with MIC values of 8, 16, 24 and 16 mg/mL, respectively [21]. In comparison, however, Chao and Yin [26] recorded much lower MIC values (24–44 µg/mL) for the protocatechuic acid against *S*. Typhimurium, *E*. *coli*, *L*. *monocytogenes*, *S. aureus* and *B*. *cereus*. Since both studies used a pure commercial compound, the difference in MIC values was expected to be small, especially because they included a bacterial strain of the same genus and species (*S*. *aureus*). In this study, hibiscus acid showed MIC values (Table 6) within the limits of those reported for protocatechuic acid [26,47]).

Finally, the anti-microbial effect of the acetone extract and hibiscus acid from *H. sabdariffa* calyces was determined as bactericidal or bacteriostatic. A compound is considered bactericidal when the MBC/MIC ratio is ≤4 and bacteriostatic when this ratio is >4 [48]. Both the acetone extract and the hibiscus acid were bactericidal against all eight multidrug-resistant pathogenic strains (Table 6). Abdallah [49] also reported the bactericidal activity of methanolic extract from *H*. *sabdariffa* calyces (MBC/MIC 1–2 mg/mL) against *A*. *baumannii* strains.

### 3.7. Measurement of Permeability with Crystal Violet

Hydrophobic crystal violet is known to display weak penetration of the outer membrane but on the contrary, it is found to penetrate cells with impaired cell membranes thus, crystal violet assay may be employed for the detection of membrane damage [38,50].

**Figure 5:**
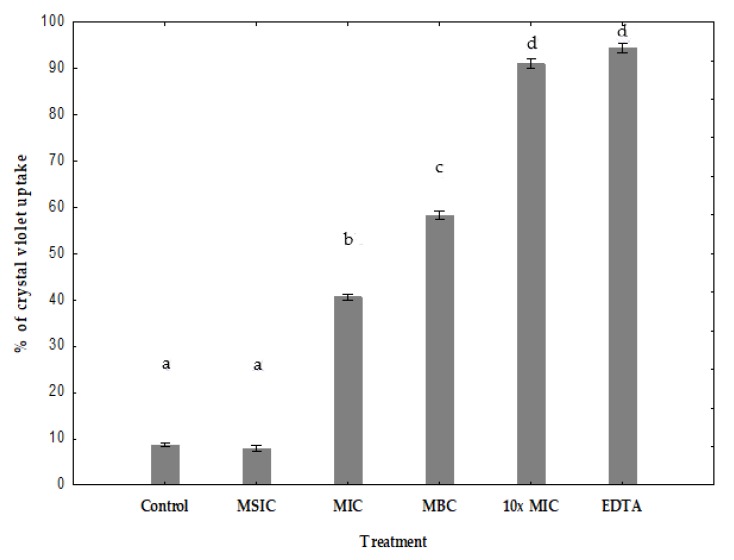
Change in bacterial membrane permeability of EHEC A (assayed by crystal violet uptake) in presence of different concentrations of hibiscus acid and EDTA. Percentage of crystal violet uptake was plotted against the concentration of the treatment. The mean ± standard deviation for three replicates are illustrated. Values with different letters express significant difference at *α* = 0.05 by Tukey’s test. MSIC: minimum sub-inhibitory concentration, MIC: minimum inhibitory concentration, MBC: minimum bactericidal concentration, 10× MIC: 10× minimum inhibitory concentration, EDTA: ethylenediaminetetraacetic acid.

**Figure 6:**
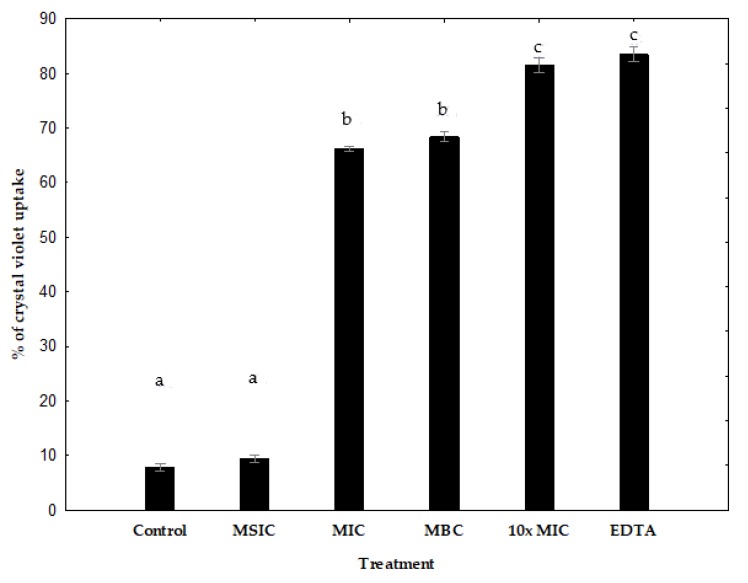
Change in bacterial membrane permeability of *Salmonella* C65 (assayed by crystal violet uptake) in presence of different concentrations of hibiscus acid and EDTA. Percentage of crystal violet uptake was plotted against the concentration of the treatment. The mean ± standard deviation for three replicates are illustrated. Values with different letters express significant difference at *α* = 0.05 by Tukey’s test. MSIC: minimum sub-inhibitory concentration, MIC: minimum inhibitory concentration, MBC: minimum bactericidal concentration, 10× MIC: 10× minimum inhibitory concentration, EDTA: ethylenediaminetetraacetic acid.

The uptake of crystal violet by enterohemorrhagic *E*. *coli* EHEC A was 9% in the absence of hibiscus acid, but increased to 40%, 57% and 90% after MIC, MBC, 10× MIC hibiscus acid treatments, respectively (Figure 5). The uptake of crystal violet by *Salmonella* C65 was 8% in the absence of hibiscus acid, but increased to 66%, 68% and 82% after MIC, MBC, 10× MIC hibiscus acid treatments, respectively (Figure 6). Minimum sub-inhibitory concentration (MSIC) of hibiscus acid showed no effect, which reveals that it did not alter the membrane permeability in both pathogenic bacteria (Figure 5 and Figure 6).

The results of the crystal violet absorption assay indicate that hibiscus acid alters membrane permeability of enterohemorrhagic *E*. *coli* EHEC A and *Salmonella* C65.

The effect of hibiscus acid on outer membrane permeability was shown by the uptake of the dye crystal violet. Crystal violet penetrates the outer membrane poorly, but it easily enters when the membrane is damaged. A significant enhancement in the uptake of crystal violet was observed in enterohemorrhagic *E*. *coli* EHEC A and *Salmonella* C65 treated with hibiscus acid when compared to control cells. This shows that hibiscus acid alters membrane permeability and makes the cells perpermeable to solutes. Furthermore, EDTA also significantly increased the uptake of crystal violet into the cells (Figure 5 and Figure 6). In Gram-negative bacteria, EDTA induces outer membrane permeabilization and cell lysis [51].

## 4. Conclusions

The present work showed that hibiscus acid is one of the compounds responsible for the anti-microbial effect of *H*. *sabdariffa* calyces. It was found at a level of 1.3% (13 g/kg) in dried *H*. *sabdariffa* calyces, and 28.3% in the dry acetonic extract from *H. sabdariffa* calyces, respectively. Due to its relatively high concentration, hibiscus acid is likely one of the main bactericidal compounds in *H. sabdariffa* calyces, although other anti-microbial compounds yet to be reported may also contribute to this effect. Both the hibiscus acid and the acetonic extract from *H. sabdariffa* constitute a potential alternative in the control of multidrug-resistant pathogenic bacteria, such as *Salmonella* and *E*. *coli* pathotypes. In addition, the hibiscus acid from *H. sabdariffa* calyces is potentially useful in the food industries given its relative abundance and availability. Finally, further research is needed to identify other anti-microbial compounds in *H*. *sabdariffa* and their mechanisms of action against bacteria. In addition, hibiscus acid affected membrane permeability of enterohemorrhagic *E*. *coli* EHEC A and *Salmonella* C65.

## Figures and Tables

**Table 1:** Chromatography fractions obtained from *Hibiscus sabdariffa* calyx acetonic extract.

Fraction Number	Solvent Ratio Used in Chromatography Column as Mobile Phase
1–37	Hexane
38–59	90–10% Hexane–ethyl acetate
60–131	80–20% Hexane–ethyl acetate
132–277	70–30% Hexane–ethyl acetate
278–348	60–40% Hexane–ethyl acetate
349–396	50–50% Hexane–ethyl acetate
397–441	40–60% Hexane–ethyl acetate
442–486	30–70% Hexane–ethyl acetate
487–535	20–80% Hexane–ethyl acetate
536–572	10–90% Hexane–ethyl acetate
573–616	Ethyl acetate
617–660	90–10% Ethyl acetate–methanol
661–693	80–20% Ethyl acetate–methanol
694–731	70–30% Ethyl acetate–methanol
732–771	60–40% Ethyl acetate–-methanol
772–794	50–50% Ethyl acetate–methanol
795–810	40–60% Ethyl acetate–methanol
811–842	30–70% Ethyl acetate–methanol
843–868	20–80% ethyl acetate–methanol
869–886	10–90% ethyl acetate–methanol
887–903	Methanol

**Table 2:** Fraction collections from *Hibiscus sabdariffa* calyx acetonic extract classified according to thin-layer chromatography.

Collection	Fraction	Collection	Fraction
I	1–42	XIV	285–379
II	43–46	XV	380–407
III	47–59	XVI	408–447
IV	60–62	XVII	448–473
V	63–68	XVIII	474–564
VI	69–107	XIX	565–584
VII	108–116	XX	585–620
VIII	117–132	XXI	621–695
IX	133–155	XXII	696–740
X	156–176	XXIII	741–792
XI	180–200	XXIV	793–867
XII	201–256	XXV	868–903
XIII	257–284		

**Table 3:** Anti-microbial effect of chromatographic fraction collections from *Hibiscus sabdariffa* calyx acetonic extract against eight multidrug-resistant *Salmonella* and pathogenic *Escherichia coli* bacteria.

Collection	*Salmonella* C1	*Salmonella* C65	*Salmonella* C63	EHEC A	EIEC MAC B	*E. coli* C558	*E. coli* C636	EPEC MAC A
VI ^1^	7.0 ± 0.2 ^b, 2^	7.2 ± 0.4 ^a^	- ^a^	- ^a^	- ^a^	- ^a^	- ^a^	- ^a^
VII	- ^a^	7.5 ± 0.1 ^ab^	- ^a^	- ^a^	- ^a^	- ^a^	- ^a^	- ^a^
IX	10.8 ± 0.2 ^g^	12.6 ± 0.2 ^gh^	13.3 ± 0.2 ^i^	11.9 ± 0.2 ^gh^	12.2 ± 0.3 ^h^	9.3 ± 0.2 ^bcd^	10.1 ± 0.5 ^efg^	12.0 ± 0.4 ^h^
X	13.5 ± 0.4 ^h^	11.5 ± 0.4 ^f^	13.3 ± 0.2 ^i^	12.3 ± 0.2 ^h^	11.6 ± 0.6 ^fgh^	11.5 ± 0.2 ^g^	11.6 ± 0.7 ^h^	12.4 ± 0.2 ^h^
XI	13.5 ± 0.2 ^h^	11.6 ± 0.2 ^fg^	15.2 ± 0.1 ^j^	14.2 ± 0.2 ^i^	12.5 ± 0.4 ^h^	11.5 ± 0.3 ^g^	13.2 ± 0.3 ^i^	11.5 ± 0.2 ^gh^
XII	11.1 ± 0.6 ^g^	12.6 ± 0.2 ^gh^	10.1 ± 0.1 ^defgh^	9.6 ± 0.5 ^ef^	10.5 ± 0.6 ^de^	11.8 ± 0.3 ^g^	10.2 ± 0.2 ^efg^	9.5 ± 0.5 ^cde^
XIII	10.5 ± 0.3 ^fg^	15.2 ± 0.5 ^i^	11.0 ± 0.1 ^h^	11.5 ± 0.3 ^g^	11.8 ± 0.5 ^gh^	11.4 ± 0.3 ^fg^	10.9 ± 0.1 ^gh^	11.0 ± 0.3 ^fg^
XIV	10.9 ± 0.4 ^g^	11.9 ± 0.5 ^fg^	10.7 ± 0.6 ^gh^	10.2 ± 0.3 ^f^	10.5 ± 0.1 ^def^	11.5 ± 0.4 ^g^	9.8 ± 0.4 ^def^	10.3 ± 0.5 ^def^
XV	10.8 ± 0.4 ^g^	13.5 ± 0.2 ^h^	10.5 ± 0.3 ^fgh^	9.5 ± 0.3 ^ef^	11.1 ± 0.5 ^efg^	9.6 ± 0.5 ^cde^	10.6 ± 0.4 ^fg^	9.6 ± 0.1 ^cde^
XVI	9.5 ± 0.1 ^def^	9.7 ± 0.4 ^e^	9.8 ± 0.4 ^defg^	9.9 ± 0.1 ^f^	10.1 ± 0.1 ^bcde^	10.4 ± 0.3 ^ef^	9.6 ± 0.4 ^de^	10.4 ± 0.1 ^ef^
XVII	9.1 ± 0.2 ^cde^	9.7 ± 0.3 ^e^	9.5 ± 0.4 ^de^	10.0 ± 0.4 ^f^	10.3 ± 0.7 ^cde^	9.7 ± 0.1 ^cde^	9.8 ± 0.4 ^def^	9.4 ± 0.7 ^cd^
XVIII	9.4 ± 0.6 ^de^	9.6 ± 0.3 ^e^	9.6 ± 0.5 ^def^	9.1 ± 0.2 ^de^	9.9 ± 0.6 ^bcd^	10.1 ± 0.7 ^de^	9.5 ± 0.1 ^de^	8.8 ± 0.2 ^c^
XIX	8.7 ± 0.2 ^cde^	8.5 ± 0.7 ^bcd^	9.3 ± 0.3 ^cd^	8.4 ± 0.3 ^cd^	9.5 ± 0.4 ^bcd^	9.3 ± 0.2 ^bcd^	7.4 ± 0.5 ^b^	7.8 ± 0.1 ^b^
XX	8.9 ± 0.4 ^cde^	9.5 ± 0.0 ^de^	9.9 ± 0.1 ^defg^	9.0 ± 0.2 ^de^	9.3 ± 0.1 ^bc^	9.2 ± 0.3 ^bcd^	8.2 ± 0.1 ^bc^	8.7 ± 0.1 ^bc^
XXI	8.1 ± 0.1 ^c^	8.8 ± 0.3 ^cde^	8.3 ± 0.2 ^b^	7.8 ± 0.2 ^bc^	9.0 ± 0.1 ^b^	8.3 ± 0.3 ^b^	8.5 ± 0.4 ^c^	8.9 ± 0.4 ^c^
XXII	8.5 ± 0.4 ^cd^	8.3 ± 0.2 ^bc^	8.6 ± 0.2 ^bc^	7.5 ± 0.2 ^b^	- ^a^	- ^a^	- ^a^	10.0 ± 0.2 ^de^
XXIII	9.6 ± 0.5 ^ef^	9.1 ± 0.4 ^cde^	10.3 ± 0.2 ^efgh^	8.6 ± 0.1 ^d^	9.0 ± 0.7 ^b^	8.9 ± 0.5 ^bc^	9.1 ± 0.1 ^cd^	10.1 ± 0.3 ^def^

^1^ Chromatographic collections showing no effect against any microorganism are not in the table. ^2^ Mean ± standard deviation of three replicas of zone of inhibition diameter (mm). - No anti-microbial effect, values with different letters in the same column per pathogen express significant difference at *α* = 0.05 by Tukey’s test. *Salmonella* C1 = *S*. Montevideo, *Salmonella* C65 = *S*. Typhimurium, *Salmonella* C63 = *S*. Typhimurium, EHEC A = enterohemorrhagic *E. coli*, EIEC MAC B = enteroinvasive *E*. *coli*, *E*. *coli* C558 and C636 = Shiga toxin-producing *E*. *coli*, EPEC MAC A = enteropathogenic *E*. *coli*.

**Table 4:** X-ray spectroscopy details of crystal data and structure refinement parameters of hibiscus acid isolated from Hibiscus sabdariffa calyx acetonic extract.

Experimental Data	
Empirical Formula	C_6_H_6_O_7_ • H_2_O
Molecular weight	208.12
Temperature (K)	293(2)
Crystal system, space group	orthorhombic, *P*2_1_2_1_2_1_
Unit cell dimensions (Å, °)	
*a*	8.2069(2)
*b*	9.9228(2)
*c*	10.1747(2)
*α(°)*	90
*β(°)*	90
*γ(°)*	90
Volume (Å^3^)	828.58(3)
*Z*	5
Radiation type	CuK*α* (*λ* = 1.54184 Å)
*μ* (mm^−1^)	1.797
*ρ*_calc_ (g cm^−3^)	2.096
*F* (000)	545.00
2*θ* range for data collection	12.46–155.038
Index Ranges	−10 ≤ *h* ≤ 10, −11 ≤ *k* ≤ 12, −11 ≤ *l* ≤ 12
Absorption Correction	Multi-scan
Collected Reflections	11147
Independent Reflections	1754 (*R*_int_ = 0.0293)
Data/Restraints/Parameters	1754/0/133
Goodness-of-fit on *F*^2^	1.077
*R*_1_, *wR*_2_ [*I* ≥ *σ*2s(*I*)]	0.0309, 0.0859
*R*_1_, *wR*_2_ [all data]	0.0314, 0.0864
Largest Difference Peak/Hole (e Å^−3^)	0.25 and −0.21
Flack and Hooft Parameters	0.05(6) and 0.07(5)
Inverted Flack and Hooft Parameters	0.95(6) and 0.93(5)

**Table 5:** Zone of inhibition diameter of *Hibiscus sabdariffa* calyx acetonic extract and hibiscus acid against multidrug-resistant *Salmonella* and pathogenic *Escherichia coli* strains.

Bacteria	Treatment	
	Acetone extract	Hibiscus Acid
*Salmonella* C1 ^1^	12.6 ± 0.1 ^a^	16.0 ± 0.4 ^b^
*Salmonella* C65	10.8 ± 0.3 ^a^	14.5 ± 0.1 ^b^
*Salmonella* C63	10.3 ± 0.3 ^a^	11.6 ± 0.2 ^b^
EHEC A	10.7 ± 0.4 ^a^	10.0 ± 0.3 ^a^
EIEC MAC B	11.5 ± 0.1 ^a^	13.4 ± 0.6 ^b^
*E. coli* C558	11.8 ± 0.1 ^a^	11.6 ± 0.4 ^a^
*E. coli* C636	10.4 ± 0.5 ^a^	11.1 ± 0.2 ^a^
EPEC MAC A	9.8 ± 0.1 ^a^	10.5 ± 0.3 ^b^

^1^ Mean ± standard deviation of three replicas of zone of inhibition diameter (mm). Values with different letters in the same row per pathogen express significant difference at *α* = 0.05 by Tukey’s test. *Salmonella* C1 = *S*. Montevideo, *Salmonella* C65 = *S*. Typhimurium, *Salmonella* C63 = *S*. Typhimurium, EHEC A = enterohemorrhagic *E*. *coli*, EIEC MAC B = enteroinvasive *E*. *coli*, *E*. *coli* C558 and C636 = Shiga toxin-producing *E*. *coli*, EPEC MAC A = enteropathogenic *E*. *coli*. Final dose per disk: 2 mg.

**Table 6:** Minimum inhibitory concentration (MIC), minimum bactericidal concentration (MBC) and MBC/MIC ratio of *Hibiscus sabdariffa* calyx acetonic extract and hibiscus acid on multidrug-resistant *Salmonella* and pathogenic *Escherichia coli* strains.

Bacteria	Acetone Extract			Hibiscus Acid		
	MIC (mg/mL)	MBC (mg/mL)	MIC/MBC	MIC (mg/mL)	MBC (mg/mL)	MBC/MIC
*Salmonella* C1	7	10	1.4	4	5	1.3
*Salmonella* C65	7	7	1.0	7	7	1.0
*Salmonella* C63	7	10	1.4	5	7	1.4
EHEC A	7	10	1.4	5	7	1.4
EIEC MAC B	7	10	1.4	5	7	1.4
*E. coli* C558	7	10	1.4	5	7	1.4
*E. coli* C636	7	10	1.4	5	5	1.0
EPEC MAC A	7	10	1.4	4	7	1.8

*Salmonella* C1 = *S*. Montevideo, *Salmonella* C65 = *S*. Typhimurium, *Salmonella* C63 = *S*. Typhimurium, EHEC A = enterohemorrhagic *E*. *coli*, EIEC MAC B = enteroinvasive *E*. *coli*, *E*. *coli* C558 and C636 = Shiga toxin-producing *E*. *coli*, EPEC MAC A = enteropathogenic *E*. *coli*.

## Supplementary Materials

The following are available online at https://www.mdpi.com/2079-6382/8/4/218/s1, Table S1: Fractional Atomic Coordinates (×104) and Equivalent Isotropic Displacement Parameters (Å2 × 103) for hibiscus acid, Table S2: Anisotropic Displacement Parameters (Å2 × 103) for hibiscus acid, Table S3: Bond Lengths for hibiscus acid, Table S4: Bond Angles for hibiscus acid, Table S5: Hydrogen Atom Coordinates (Å × 104) and Isotropic Displacement Parameters (Å2 × 103) for hibiscus acid.
