# Supplementary material for: Hibiscus Acid and Chromatographic Fractions from Hibiscus Sabdariffa Calyces: Antimicrobial Activity against Multidrug-Resistant Pathogenic Bacteria
Source: Antibiotics (Basel). 2019 Nov 11;8(4):218. doi: 10.3390/antibiotics8040218 (PMC6963829; doi:10.3390/antibiotics8040218)
Supplement: Supplementary file 1 [file antibiotics-08-00218-s001.pdf]

antibiotics

Table S1. Fractional Atomic Coordinates (×10⁴) and Equivalent Isotropic Displacement Parameters (Å²×10³) for hibiscus acid.

Atom	*x*	*y*	*z*	U_{eq}*
C1	6594 (2)	4917.2 (17)	6499.1 (17)	23.9 (4)
C2	6908 (2)	6431.9 (17)	6560 (2)	30.8 (4)
C3	8725 (2)	6546.3 (19)	6369.0 (19)	28.4 (4)
C4	8250 (2)	4373.5 (17)	7028.1 (18)	25.3 (4)
C5	8675 (2)	3024.4 (18)	6426.0 (19)	29.0 (4)
C6	5129 (2)	4537.5 (17)	7348.9 (18)	26.3 (4)
O1	9447.7 (16)	5366.2 (14)	6649.7 (14)	30.9 (3)
O2	9497 (2)	7515.4 (15)	6041.3 (16)	39.5 (4)
O3	6340.5 (17)	4575.5 (14)	5179.5 (12)	30.4 (3)
O4	7534 (2)	2148.1 (14)	6752.0 (19)	41.5 (4)
O5	9855 (2)	2804.7 (16)	5765.9 (17)	43.1 (4)
O6	5410.3 (17)	4662 (2)	8607.0 (14)	40.2 (4)
O7	3841.3 (18)	4209.0 (17)	6882.1 (15)	38.3 (4)
O8	2424.9 (19)	4810.5 (15)	9551.0 (14)	34.6 (3)

*U_{eq} is defined as 1/3 of the trace of the orthogonalised U_{IJ} tensor.

Table S2. Anisotropic Displacement Parameters (Å² × 10³) for hibiscus acid. *.

Atom	U_{11}	U_{22}	U_{33}	U_{23}	U_{13}	U_{12}
C1	22.9 (7)	21.7 (7)	27.1 (8)	−0.6 (6)	−0.8 (6)	0.9 (6)
C2	27.4 (9)	21.2 (8)	43.9 (10)	−1.4 (7)	−1.3 (8)	0.1 (7)
C3	30.2 (9)	25.4 (8)	29.6 (9)	−3.5 (7)	−0.8 (7)	−2.4 (7)
C4	22.9 (7)	24.6 (8)	28.4 (8)	−1.0 (6)	0.5 (7)	−0.1 (7)
C5	29.3 (9)	23.9 (8)	33.7 (9)	0.4 (7)	−1.5 (7)	4.1 (7)
C6	24.7 (8)	22.7 (8)	31.5 (8)	0.9 (6)	0.0 (7)	1.5 (7)
O1	23.2 (6)	27.5 (6)	41.9 (7)	−1.6 (6)	0.2 (5)	−1.3 (5)
O2	37.5 (7)	28.7 (7)	52.3 (9)	1.4 (6)	2.4 (7)	−9.2 (6)
O3	32.7 (7)	31.6 (7)	27.1 (6)	0.9 (5)	−1.2 (5)	−7.5 (6)
O4	39.8 (8)	24.8 (6)	60.0 (9)	−6.0 (7)	7.0 (7)	−1.9 (6)
O5	41.0 (8)	33.7 (8)	54.5 (9)	−8.8 (7)	13.3 (7)	6.0 (7)
O6	26.6 (6)	65.8 (10)	28.3 (7)	−2.9 (7)	2.3 (5)	−0.4 (7)
O7	28.6 (7)	50.6 (9)	35.7 (7)	5.6 (6)	−3.0 (6)	−11.4 (6)
O8	33.3 (7)	33.3 (7)	37.1 (7)	−4.3 (6)	10.4 (6)	−2.3 (6)

* The Anisotropic displacement factor exponent takes the form: $-2\pi^2 [h^2a^{*2}U_{11}+2hka^*b^*U_{12}+\ldots]$.

Table S3. Bond Lengths for hibiscus acid.

Atom	Atom	Length/Å
C1	C2	1.526 (2)
C1	C4	1.558 (2)
C1	C6	1.528 (2)
C1	O3	1.400 (2)
C2	C3	1.508 (3)
C3	O1	1.343 (2)
C3	O2	1.199 (3)
C4	C5	1.513 (2)
C4	O1	1.444 (2)
C5	O4	1.320 (3)
C5	O5	1.198 (3)
C6	O6	1.307 (2)
C6	O7	1.203 (2)

Table S4. Bond Angles for hibiscus acid.

Atom	Atom	Atom	Angle (°)
C2	C1	C4	100.33 (14)
C2	C1	C6	110.66 (14)
C6	C1	C4	113.93 (14)
O3	C1	C2	107.61 (15)
O3	C1	C4	112.14 (14)
O3	C1	C6	111.46 (14)
C3	C2	C1	103.65 (15)
O1	C3	C2	110.10 (15)
O2	C3	C2	128.21 (19)
O2	C3	O1	121.68 (18)
C5	C4	C1	111.56 (14)
O1	C4	C1	105.39 (13)
O1	C4	C5	109.81 (14)
O4	C5	C4	108.51 (15)
O5	C5	C4	125.04 (18)
O5	C5	O4	126.46 (18)
O6	C6	C1	113.07 (16)
O7	C6	C1	122.30 (17)
O7	C6	O6	124.57 (18)
C3	O1	C4	110.54 (14)

Table S5. Hydrogen Atom Coordinates (Å × 10^4) and Isotropic Displacement Parameters (Å^2 × 10^3) for hibiscus acid.

Atom	*x*	*y*	*z*	U_{eq}*
H2A	6325	6900	5868	37
H2B	6582	6799	7404	37
H4A	8210	4296	7988	30
H3	5802	3881	5138	46
H4	7739	1416	6417	62
H6	4551	4592	9013	60
H8A	2171	5074	10319	52
H8B	1785	4182	9315	52

*U_{eq} is defined as 1/3 of the trace of the orthogonalised U_{IJ} tensor.
